# Live Intersection Data Acquisition for Traffic Simulators (LIDATS)

**DOI:** 10.3390/s24113392

**Published:** 2024-05-24

**Authors:** Andrew Renninger, Sinan Ameen Noman, Travis Atkison, Jonah Sussman

**Affiliations:** Computer Science, College of Engineering, University of Alabama, Tuscaloosa, AL 35401, USA; sanoman@crimson.ua.edu (S.A.N.); atkison@cs.ua.edu (T.A.); jasussman3@crimson.ua.edu (J.S.)

**Keywords:** digital twin, intelligent transportation systems, traffic signal controller, data acquisition, traffic simulation

## Abstract

Real-time traffic signal acquisition and network transmission are essential components of intelligent transportation systems, facilitating real-time traffic monitoring, management, and analysis in urban environments. In this paper, we introduce a comprehensive system that incorporates live traffic signal acquisition, real-time data processing, and secure network transmission through a combination of hardware and software modules, called LIDATS. LIDATS stands for Live Intersection Data Acquisition for Traffic Simulators. The design and implementation of our system are detailed, encompassing signal acquisition hardware as well as a software platform that is used specifically for real-time data processing. The performance evaluation of our system was conducted by simulation in the lab, demonstrating its capability to reliably capture and transmit data in real time, and to effectively extract the relevant information from noisy and complex traffic data. Supporting a variety of intelligent transportation applications, such as real-time traffic flow management, intelligent traffic signal control, and predictive traffic analysis, our system enables remote data analysis and decisionmaking, providing valuable insights and enhancing the traffic efficiency while reducing the congestion in urban environments.

## 1. Introduction

Intelligent transportation systems (ITSs) encompass a rapidly growing field. The main goal of ITSs is to improve transportation as well as make the system safer, more reliable, and more efficient without altering the existing infrastructure physically [1]. ITSs represent a new global phenomenon that benefits the public as well as private sectors. For instance, they help in managing several government policies and making procedures feasible, such as transportation safety compliance, customs clearance safety, as well as toll collection. Additionally, they enhance corporate productivity by reducing operating costs, producing time savings, and helping in saving energy [2].

A significant advancement in the field of ITSs is digital twin technology, which creates virtual replicas of physical systems—for example, traffic networks—enabling simulation, monitoring, and proactive control over time in near real time. A digital twin will be a central component as it plays a vital role in optimizing traffic flow, increasing safety, and reducing traffic congestion. This is achieved by facilitating the rigorous testing and refinement of the strategic innovation within the virtual world prior to the real-world implementation.

In ITSs, data aggregation is considered to be one of the crucial aspects. They gather data from various sources to provide holistic knowledge for the transportation system.

Another crucial aspect of ITSs includes traffic signal controllers. While these controllers can primarily manage the timing and operation of traffic signals, it is the sensors associated with these controllers that gather the data on traffic flow, volume, and density. These data are essential when analyzing transportation patterns [3]. Furthermore, they combine the information that is gathered from various traffic signal controllers and provide a comprehensive understanding of the transportation system in a given area.

Aggregating data from traffic signal controllers requires using specialized software and hardware to collect the data in real time. Generally, the hardware devices, which include various sensors, are installed at intersections. These sensors are capable of gathering various types of data related to the traffic, such as vehicle speed, traffic flow, and other metrics, which are then utilized by the traffic signal controllers to manage the signals effectively. On the other hand, the specialized software can be used to aggregate and analyze the collected data from the traffic signal controllers. For instance, Geographic Information Systems (GIS) can map the patterns of traffic, while traffic simulation software models various traffic scenarios. Traffic management systems, on the other hand, can optimize signal timings based on real-time data [4].

Analyzing aggregated data paves the way for each of the Department of Transportation (DOT) levels—federal, state, and local—to find the congestion areas, as well as to develop targeted traffic management strategies. The DOT can implement data-driven solutions with ITS insights, such as adaptive traffic signal control, real-time traffic information, and smart parking systems, to reduce delays as well as to enhance the traffic flow. These approaches play a vital role in improving the transportation safety, efficiency, and reliability across all the DOT jurisdictions. They also contribute to improving the infrastructure planning and policy-making.

**Figure 1:**
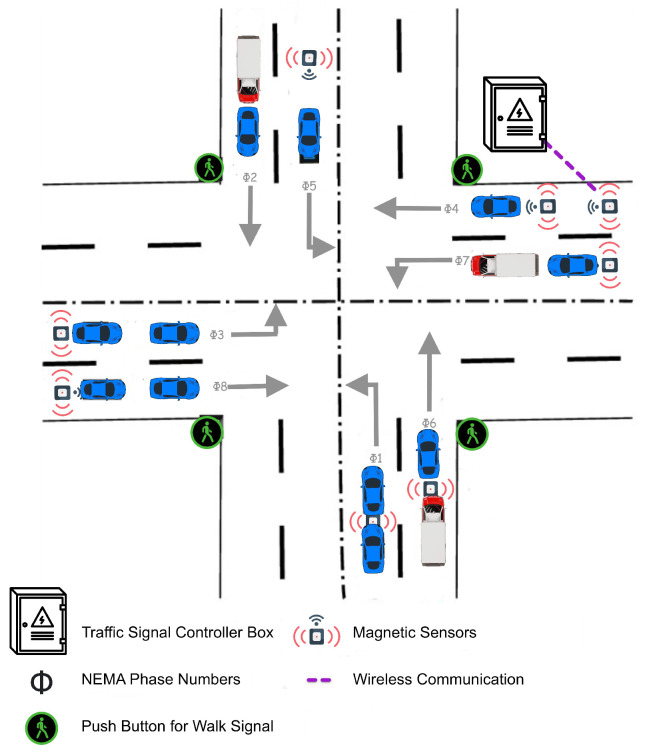
Key components for traffic data collection.

Figure 1 illustrates the components involved in the data aggregation process, which includes the National Electrical Manufacturers Association (NEMA) phase numbers, a pedestrian push button for walk signals, a traffic signal box, and magnetic sensors. NEMA is a standardized numbering scheme for traffic signal phases that ensures the consistency and interoperability among traffic signal controllers. Generally, there are eight phase numbers assigned to the main and side streets. This helps traffic engineers to design and manage coordination plans as well as signal timings efficiently [5].

As shown in Figure 1, the magnetic sensor transmits a signal wirelessly to the traffic signal controller when the vehicle passes over it, providing near real-time information on the number of vehicles currently in the area corresponding to each NEMA phase. This information is very important as it helps in understanding the real-time traffic situation at the intersection. Also, the pedestrian push button transmits a message to the traffic signal box every time it is pressed. It indicates that a change has happened in the walk signal status. These data play a vital role in optimizing the signal timings for pedestrian safety, as well as making the traffic flow more efficient. In this paper, it is essential to note that the focus of this study is on demonstrating how our system is capable of aggregating and handling the data from traffic signal controllers, not on the efficiency or optimization of the traffic phases depicted. However, the data that we collect can be used to improve efficiency.

As stated earlier, this paper focuses explicitly on the data aggregation process in ITSs, particularly on the data obtained from traffic signal controllers. Although data aggregation can be facilitated by manufacturer solutions, a unified approach that is capable of aggregating data in real time and compatible with a variety of traffic simulators is not prevalent. Traffic simulators are software tools that are used to model the behavior of traffic systems; they predict the traffic flow and also assess the impact of various traffic management strategies. While improvements in traffic control algorithms have used simulations of intersections [6,7], they could benefit from these real-time data.

To bridge the gap, this paper presents LIDATS (Live Intersection Data Acquisition for Traffic Simulators), an architecture that aims to provide a real-time data stream for traffic simulators from traffic signal controllers. Our goal is to ensure that the stream is secure, quick, and consumes low bandwidth.

The recent findings demonstrate a gap in ITSs regarding aggregating data in real time. Moreover, most of the current solutions are based on proprietary hardware and software configurations, which makes it difficult to scale and adapt to different urban environments. Another critical limitation observed in the existing digital twin technologies is the lack of a real-time connection between virtual simulations and on-site traffic control systems. This gap reduces the effectiveness of digital twins in ITSs as it limits their use when it comes to making quick decisions and managing traffic actively.

In order to overcome these limitations, a unified approach is needed that is capable of enabling the seamless integration of various traffic management systems and real-time processing. These constraints restrict the broader application of ITSs and their efficiency in managing traffic flows and responding to real-time urban traffic conditions.

This paper contributes to the field of ITSs by introducing the LIDATS system, which provides several advances:

- Comprehensive System Architecture: In this paper, we detail how we developed our LIDATS system that includes robust software and hardware designed to enhance the acquisition and processing of data in real time.
- Innovative Data Acquisition and Management: We explain further the methods and technologies employed in LIDATS to ensure effective real-time handling, highlighting its advanced features.
- Support for Extensive ITS Applications: We demonstrate how our LIDATS system supports a wide range of ITS applications and how it can be used to improve the overall traffic management.

Section 2 of this paper provides a comprehensive literature review of the existing research related to our topic. In Section 3, we describe our LIDATS method and provide a detailed explanation of the hardware and software components that we used, the process of gathering data, and relevant technical specifications. Section 4 presents our evaluation, including the system uptime, latency, and data processing savings. Section 5 shows the versatile integration of LIDATS. Section 6 presents the benefits of digital twins for traffic management. Finally, in Section 7, we discuss our future work and the directions for further research based on our evaluation.

## 2. Literature Review

This literature review is organized into two subsections, each subsection representing a critical component in our LIDATS approach. The first subsection investigates the digital twin concept that involves creating a virtual replica of a system that enables the simulation and optimization of different scenarios and improves the decisionmaking and system performance. The second subsection examines the data aggregation in the context of IoT. This section aims to provide an overview of the current state of the art in ITSs by exploring the latest research and development in digital twins and data aggregation.

### 2.1. Digital Twins

A digital twin is considered as a virtual replica of a physical object, process, or system, using real-time data and simulation to provide insight and support decisionmaking. Recently, digital twin analysis has become an increasingly important and popular research area across many applications, such as manufacturing, medicine, oil, and city planning [8]. The growing number of research studies and publications on this topic and the increased attention from government sectors and industries prove this. A digital twin can provide valuable information and inform the decisionmaking in transportation by creating a virtual representation of real-world traffic systems. This subsection summarizes and analyzes the recent papers focusing on digital twins in ITSs.

Kusic et al. [9] created a digital twin of the motorways of Barcelona using traffic counters with minute real-time accuracy. There is distinct importance regarding the timing of real-time data. The data must be close to real-time in order for the simulation to be accurate. Sánchez-Vaquerizo built a large-scale microsystem of the motorways of Geneva [10] using novel mobility data from phone records and traditional surveys—not using real-time data. It is still important as a simulation of data, but, as a simulation of past data, it is more equivalent to a digital shadow. In [11], the traffic simulator SUMO was connected with the game engine Unity. It provides a 3D visualization of the simulation. This fusion enabled the SUMO to control the normal vehicles, while Unity controlled connected and automated vehicles (CAV) to achieve the efficient integration of CAV into typical traffic.

However, the existing digital twin scenarios are often lacking the data needed. In a review of digital twins for ITSs, ref. [12] concludes that data loading technology and information extraction technology still need to be strengthened. Others agree as well [9], even saying that the “Existing traffic control simulation fails to realize the real-time connection between simulation and actual traffic control on-site, making real-time simulation, verification, and evaluation unavailable” [13]. Therefore, this paper intends to provide a proven process for collecting real-time data in order to bridge the gap.

### 2.2. Data Aggregation in Internet of Things

Generally, data aggregation is a technique that involves combining and processing data from several sources to extract valuable information. It is used widely in various applications, such as transportation, healthcare, smart grid systems, etc. Examining the papers in this area provides insights into the latest developments in data aggregation, which can improve the effectiveness of our research in this area. This subsection sheds light on the recent papers discussing the different data aggregation aspects in IoT systems.

In the realm of IoT, data aggregation has experienced remarkable growth and discoveries. As early as the introduction of intelligent transportation systems, researchers like Sysoev et al. [14] investigated the possibilities of aggregating different data types to estimate traffic flow parameters. This research paper provided the foundation for the subsequent research that aimed to enhance the precision of traffic flow estimation by using multiple data sources and suitable aggregation techniques. However, the Big Data approach it takes is not always feasible due to the costs, complexity, and privacy.

Simultaneously, the IoT environment saw the emergence of inventive data aggregation approaches to tackle the challenges posed by the vast amount of data generated by IoT devices. A research paper presented a unique distributed data aggregation method [15] that aimed to improve the efficiency and dependability of IoT systems. Through experiments and simulations, Homaei et al. showcased the superiority of their proposed approach compared to the existing methods for low-power and lossy networks, opening the door for further advancements in this area.

The study of data aggregation in wireless sensor networks also made significant progress, with comprehensive reviews [16] comparing the pros and cons of various techniques, such as in-network aggregation, hierarchical aggregation, and compressive sensing-based aggregation. These contributions have emphasized the challenges of implementing data aggregation as well as spurred additional research in several areas, such as energy efficiency, data accuracy, and security.

In the field of E-health monitoring systems, Bhowmik et al. introduced an IoT-based data aggregation method [17] that revolutionized the collection of health data from multiple sources. This breakthrough enabled healthcare professionals to make informed decisions in real time, considerably improving the efficiency of such systems. Consequently, a secure aggregation algorithm [18] was proposed by Kumar et al. to balance between the privacy and energy efficiency in data aggregation, which showed tremendous promise for various applications.

As IoT systems have expanded, latency has emerged as a crucial problem. A state-of-the-art adaptive control algorithm [19] has been introduced by Yoshino et al. to minimize the latency in IoT gateways, offering a promising solution to this problem. However, it did not address non-statistical data aggregation, where the data are bundled together instead of simply summarizing the data. In the context of industrial smart grid systems, security took center stage with a novel approach [20] that combined the traditional aggregation methods with batch verification to enhance the protection of metering data.

Further research looked into the combination of node clustering and the Extreme Learning Machine (ELM) in wireless sensor networks [21] and the application of homomorphic encryption in smart grid systems [22] for secure data aggregation. These novel schemes demonstrated the importance of balancing the accuracy, communication costs, and security in the IoT network environments [23].

The field of data aggregation has indeed evolved significantly, with comprehensive overviews [24]. Cai et al. present a taxonomy of the existing data aggregation methods and design guidelines for choosing the appropriate aggregation technique for different applications. The introduction of secure data aggregation schemes, such as RESDA [25], highlights the potential of IoT applications in the healthcare domain, where data privacy and confidentiality are essential.

The evolution of data aggregation in IoT systems has been characterized by multiple studies that addressed various challenges, such as accuracy, energy efficiency, security, and privacy. These endeavors have collectively shaped the development of improving the data aggregation methods, which can be utilized in various applications, ultimately enhancing the performance of IoT systems.

## 3. LIDATS Method

**Figure 2:**
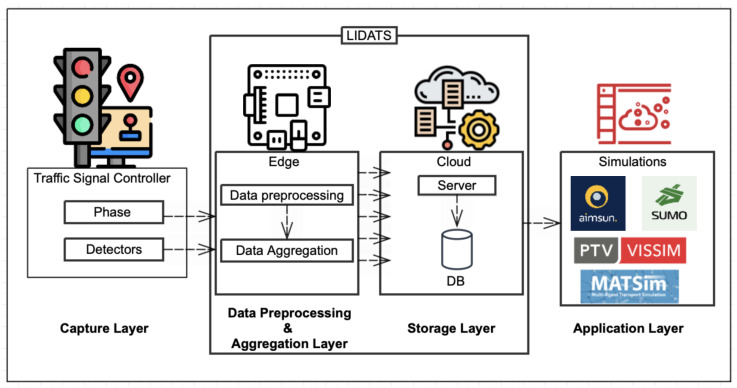
LIDATS project architecture.

This section provides a detailed overview of the LIDATS approach for real-time data acquisition from traffic signal controllers. Our approach consists of 4 layers, as shown in Figure 2. These are the capture layer, data pre-processing and aggregation layer, storage layer, and application layer.

1. The capture layer consists of two components: hardware and software. Figure 2 illustrates the architecture of LIDATS and demonstrates the process of gathering data from traffic signal controllers in real time. This layer is responsible for capturing data on the current state of the intersection from the traffic controller, accomplished through a device connected to the same local network. This is the lowest layer, and as such is the most vulnerable layer to physical interference and tampering. The data captured include the current phase, as well as data from the vehicle sensors, actuators, and pedestrian sensors or buttons. These vast data are then transferred to the data pre-processing layer.
2. The data pre-processing and aggregation layer is pivotal in refining the collected data, ensuring a balance between data integrity and efficiency. The first step is data cleaning, and removing any data that are invalid or erroneous. An example of this could be getting back data that show perpendicular directions both displaying green. Rather than discarding this information, it is annotated for further analysis, which is critical for system integrity checks and long-term improvements. In the next step, a large amount of data are sent to the data pre-processing layer, which must be carefully minimized in order to maintain the trade-off between the real-time accuracy of the data and the size and amount of data transmission. We send updates selectively, triggering transmission only when there is a notable change, like shifts in traffic lights or sensor readings. This approach prioritizes efficient data transfer, ensuring updates are conveyed in response to relevant traffic alterations. The data are then put in a minimized format and sent to the storage layer.
3. In the storage layer, the data are stored in a database, where they can be used to train machine learning models for traffic control, as well as sent to real-time traffic simulation software, in the application layer. The database can be used with traffic and crash data to determine better control algorithms for intersections and lights.
  This layer manages and addresses different types of traffic data, which are essential for managing traffic systems, including
  - Traffic Flow Data: Gathers all the relevant metrics, such as vehicle counts, speed, and density, which are useful in analyzing the patterns of the traffic as well as optimizing its flow.
  - Traffic Incident Data: Records details of traffic-related incidents, including collisions and non-collision events such as vehicle breakdowns, to provide the necessary data for safety analysis and emergency response planning.
  Generally, the traffic data are stored in advanced database management systems that facilitate efficient data retrieval and scalability, which are crucial for real-time processing and long-term traffic studies.
4. The application layer is where the real-time data are used to simulate traffic as well as predict the traffic behavior under different scenarios. For example,
  - Aimsun and SUMO: Traffic flow and incident data are utilized to model traffic simulations to test traffic management strategies.
  - MATSim: Uses traffic flow data for modeling daily travel patterns as well as optimizing routes.
  - Unity: Applies incident data in conducting detailed simulations of accidents for safety analysis.

The hardware part of our proposed approach includes an edge device; specifically, we used Raspberry Pi 4 (Raspberry Pi Foundation, Cambridge, UK) with a Linux operating system. The edge device is not equipped with any sensors. Moreover, a Hardware Security Model (HSM) is included in the proposed model to secure the data and the edge device.

The software component of the LIDATS approach is a tailor-made solution, specifically using the Simple Network Management Protocol (SNMP) [26] to gather data from the traffic signal controllers. We used the SNMPGET command and the traffic signal controller’s proprietary Object Identifier (OID) to request and gather various types of traffic data.

In the following subsections, we will describe in detail the hardware and software components as well as provide technical specifications.

### 3.1. Hardware

The hardware component of our approach involves using a Raspberry Pi 4 single-board computer with 8GB of RAM as the edge device. The Raspberry Pi, generally, is considered a low-cost and low-power device. It also has multiple interfaces, which make it a great candidate for an edge computing application. The board of the Raspberry Pi includes a 64-bit ARM (Quad-core ARM Cortex-A72) that runs at 1.5 GHz. These features play a vital role in improving the performance of the Pi compared with its predecessors. Moreover, it uses the Broadcom BCM2711 system-on-chip (SoC). The BCM2711 SoC is a single chip that integrates within it not only the CPU, GPU, and memory but also peripherals like USB, Ethernet, and Wireless, thereby making the design of the single-board computer compact [27]. The Raspberry Pi runs an operating system from the Linux family, Raspberry Pi OS (previously called Raspbian), providing a solid software environment into which Python scripts run and network communication tools are installed and configured properly for interfacing with the traffic signal controller.

Additionally, it has also been configured to interface with the traffic signal controllers, which capture signal timing, vehicle/pedestrian volume, and identify incidents at intersection. These data are invaluable for on-the-go analysis and become pre-processed on site for the maintenance of responsiveness immediacy and data integrity before being transferred to the central system for more processing.

**Figure 3:**
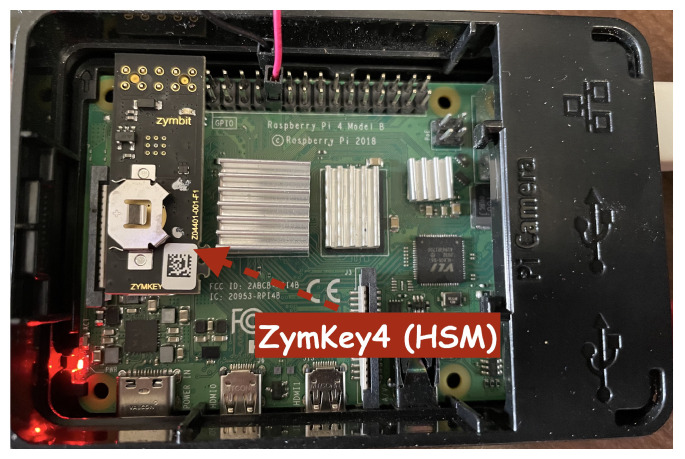
Raspberry Pi 4 with the Zymkey4 HSM module installed.

In order to enhance security, several security measures have been applied to the edge device. This includes changing the default password of the Pi as well as disabling unnecessary Pi features, and installing fail2ban for defense against brute-force attacks. Additionally, we used the Zymkey4 encryption hardware security module (HSM) by installing it on the edge device [28]. This hardware security module provides secure key storage, secure boot, and hardware-based encryption and decryption to protect the data on the edge device. This security module is designed specifically for Raspberry Pi, making it very easy for integration and into IoT projects. Additionally, it provides robust protection against several types of attacks, such as unauthorized access, theft, and data tampering. Figure 3 illustrates the Raspberry Pi 4 with the Zymkey4 HSM module installed.

**Figure 4:**
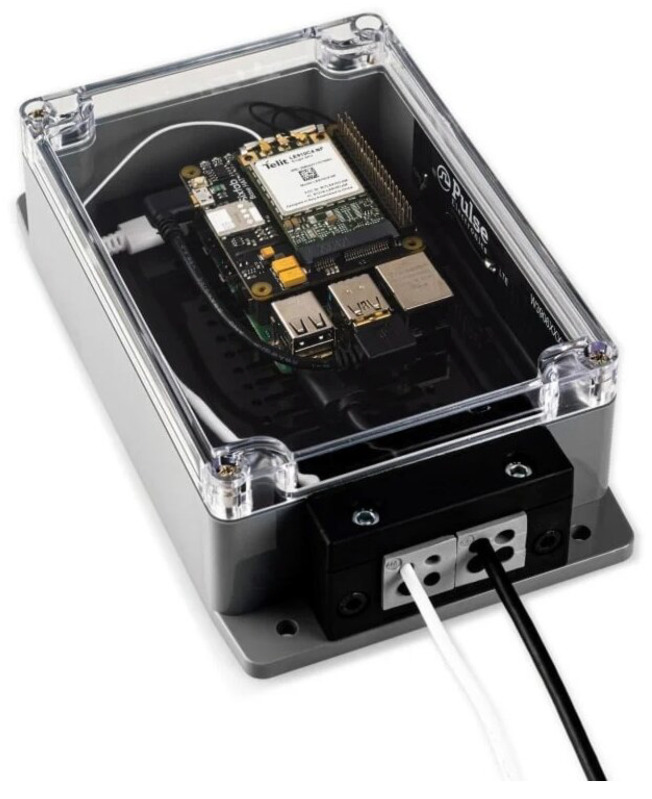
Outdoor IoT enclosure for Raspberry Pi [29].

In addition to security measures, we will use a specialized Raspberry Pi enclosure that designed to be water/weatherproof and dustproof to protect the hardware components of the Raspberry Pi and its cables. The transparent clear top cover of the enclosure has a built-in silicone gasket seal, while the base plate supports mounting any Raspberry Pi model with different orientations. Figure 4 illustrates the Raspberry Pi enclosure.

### 3.2. Software

The edge device uses SNMP, which is a protocol used for management and monitoring of network-connected devices, to poll the connected traffic controller for current state. The data retrieved are then aggregated through the LIDATS method described above. Python was utilized as the primary programming language for this purpose, chosen for its flexibility and ease of use. Authentication and preventing message forgery were the main points when considering encryption methods. We used TLSv1.3 for the encryption, authentication, speed, and message integrity [30] that it provides. We use ECDHE, Elliptic Curve Diffie–Hellman, for the key exchange. Authentication is completed through ECDSA. On the server, upon a successful handshake, the data are accepted and saved in a database.

**Figure 5:**
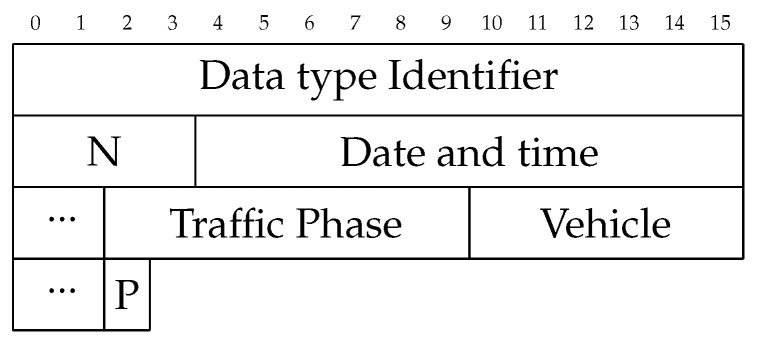
Data frame sent by edge device.

Each portion of traffic data contains at the minimum 37 bytes. A datatype identifier is first, identifying which type of data comes next. For intersection data, this consists of a 32-bit (4 byte) unsigned integer indicating the number of data points being sent, N. Following are N frames consisting of 14 bytes for the date and time, 8 bytes for the traffic phase, 64 bits (8 bytes) for the vehicle detection sensors, and 8 bits for the pedestrian detection sensors, as shown in Figure 5. Even assuming each intersection sending a single frame, every second, 500 intersections would be 17.5 Kb/s. If the connection was dropped after each frame, the most data sent would be the connection and authentication.

This system allows for more data to be sent from the intersection. Data from traffic counters like the ones from [10] could easily be added to this framework.

For storing our results, we used MongoDB, a popular NoSQL database known for its document-oriented structure and schema flexibility. MongoDB stores its data in JSON-like documents with optional schema, which complements and aligns with the flexibility of data LIDATS can capture. This allows us to adapt quickly to changing data models. Another reason for using MongoDB is its query performance at large scales: its execution time scales less than MySQL Workbench 6.3 [31].

## 4. Evaluation

The evaluation of LIDATS can be divided into system tests and digital twin readiness. We conducted five tests: four using the Econolite traffic signal controller simulator (Econolite, Anaheim, CA, USA), and one using an Econolite ASC/3-2100 to validate the effectiveness of our approach. This simulator is capable of emulating a traffic signal controller in functionality, appearance, and interface. The simulator accepts SNMPGET and SNMPSET messages and uses the same OIDs as the ASC/3-2100, allowing us to assess the performance of our method accurately.

### 4.1. System Tests

**Figure 6:**
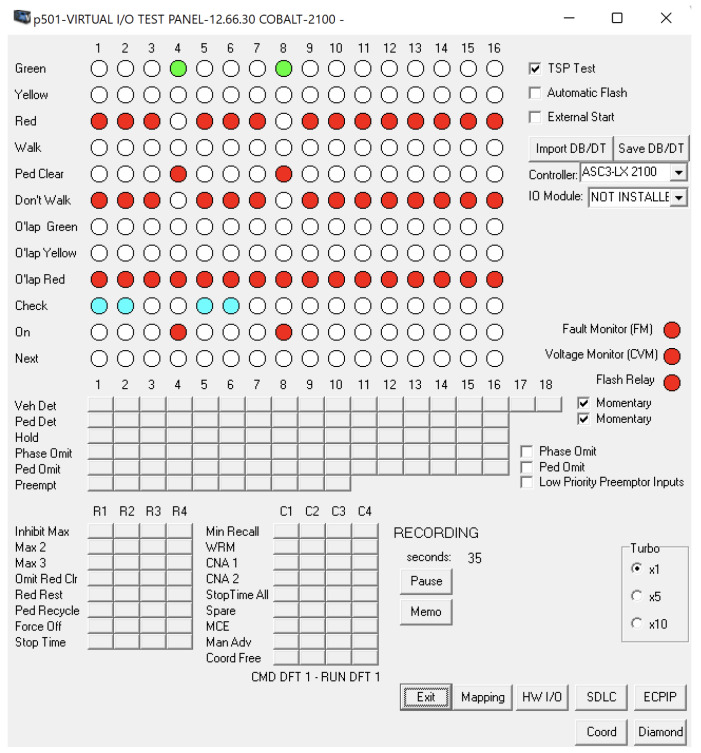
Econolite traffic signal controller simulator user interface.

Figure 6 illustrates the user interface of the Econolite traffic signal controller simulator. The simulator provides a comprehensive user interface for observing, configuring, and controlling the simulated traffic signal controller, which enabled us to evaluate our method thoroughly in various scenarios.

Using this traffic signal controller, we were able to simulate 138 intersections for over 24 h with no server issues (100% uptime). The simulated intersections were set to cycle continuously, at a faster rate than a real intersection would—at an around 60 s average cycle time. With an 120 average cycle time, this would be equivalent to 28 h. In total, over 4 tests, the simulations were polled 5,706,088 times, with a total of 2,287,240 phase changes transmitted.

Additionally, in our fifth test, we utilized an Econolite ASC/3-2100 traffic controller to test the execution of the full system. Using the same accelerated traffic cycle as before, we collected light signal data for 375 h (15.6 days), collecting 111,690 phase changes with 0 dropped transmissions, an equivalent of a entire month’s changes at a 120 average cycle time.

**Figure 7:**
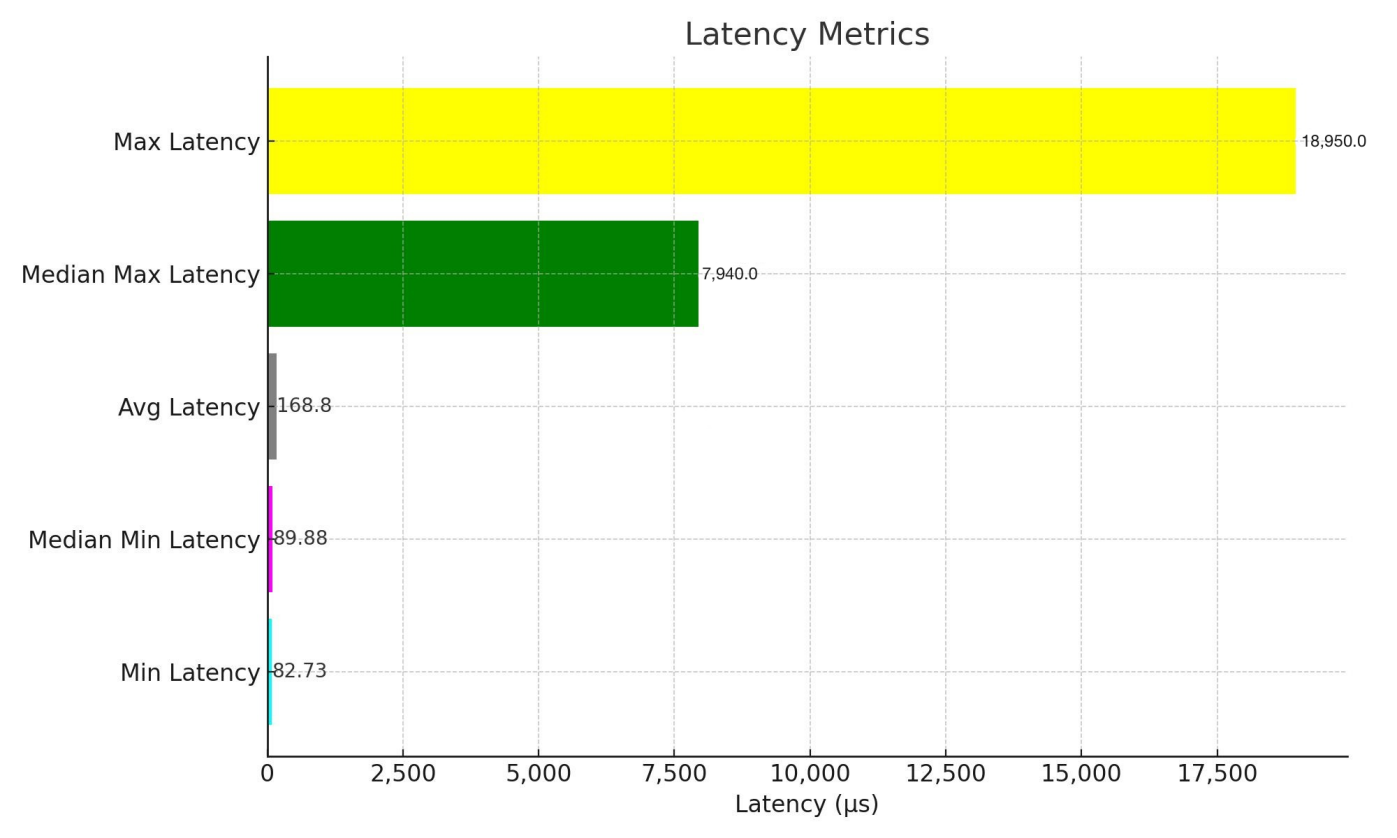
Data latency.

To measure the performance of data acquisition, we added timing commands to the edge client. A total of 80.05 megabytes of intersection data were transmitted (not including encryption), with an average latency from data acquisition to server of 0.1688 ms. The minimum latency from all intersections was 82.73 μs, with a median minimum of 89.88 μs. The maximum latency was 18.95 ms, with a median maximum of 7.94 ms, as illustrated in Figure 7.

The low latency observed in Figure 7 demonstrate the capability of LIDATS in gathering and transmitting intersection data with minimal delays. This low latency is crucial as it plays a vital role in maintaining an accurate real-time representation to simulate the traffic condition properly. Most of the data points showed that the latency was within an acceptable range for traffic simulations, which means that our LIDATS system could update frequently to reflect traffic changes accurately. Although there were a few instances of higher latency, these did not significantly affect the overall performance of LIDATS.

**Figure 8:**
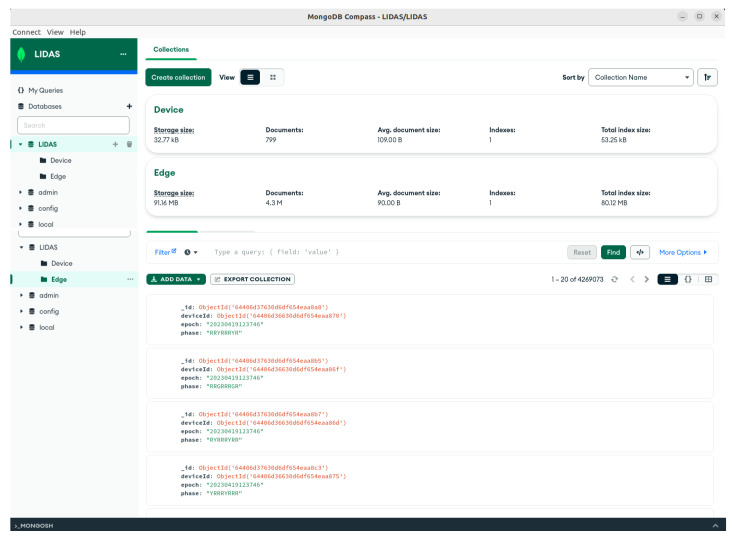
MongoDB storage and data points.

All of these data were stored in our MongoDb database, resulting in 49.26 MB on disk, as shown in Figure 8.

**Figure 9:**
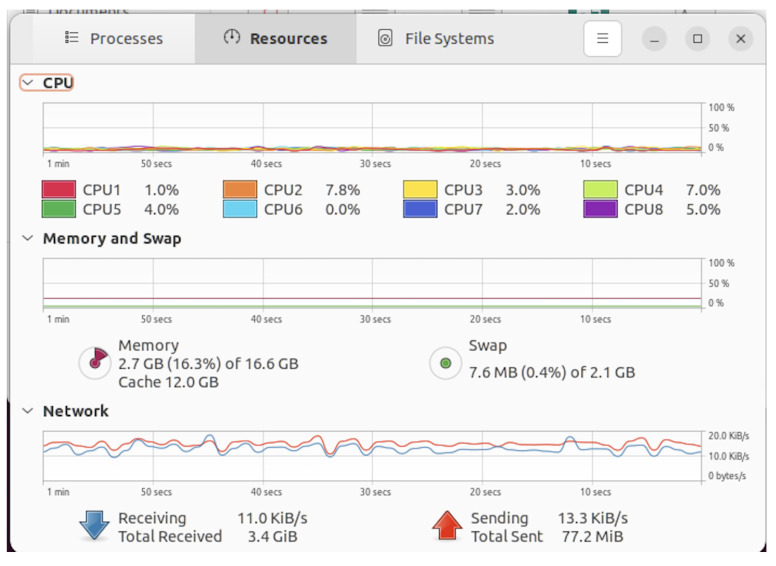
Server performance.

A consumer-grade desktop with an i7-7700 CPU running 20.04 Ubuntu Linux was very capable of capturing and storing the aggregated data, with each thread staying well under 30% usage, as shown in Figure 9.

The results of our evaluation showed that the LIDATS method successfully captured and processed real-time traffic data from the Econolite traffic signal controller simulator. The data were effectively minimized and transmitted to the aggregation layer, where they were stored in a database for further analysis and use in training machine learning models for traffic control. Additionally, the server kept track of the most recent data for each intersection to send for use in digital twin software.

### 4.2. Digital Twin Readiness

The aggregated and stored data are ready to be used in digital twin and machine learning applications alike. The database can be queried for the traffic control data for any intersection over any time length. Additionally, this traffic control information can be used to dynamically control and calibrate traffic control simulations in real time using SUMO Traffic Control Interface (TraCI). This enables simulating traffic control of intersections across cities.

## 5. LIDATS: Versatile Integration

With the successful gathering of Econolite traffic simulator data, the LIDATS architecture, therefore, is open to the possibility of wider application. This achievement suggests that LIDATS is ready to follow through to the final stage: merging with a broad-span traffic simulation platform. This capability is the flexibility of the LIDATS approach, allowing open sources from SUMO and commercial products from Aimsun, VISSIM, and PARAMICS microscopic simulation capabilities, as well as advanced capabilities from TransModeler. With the modular design, LIDATS can be easily adapted to the most varied needs of simulating traffic. This section will introduce how this integration can be accomplished with ease on these platforms. Table 1 below summarizes the integration capabilities of LIDATS with the named platforms to realize a complete digital twin in the simulation. This section will explore in detail how LIDATS can be integrated with the traffic simulation platforms.

### 5.1. Open Source Traffic Simulation: Simulation for Urban Mobility (SUMO)

SUMO is a potent open-source tool that is heavily modular and benefits from our LIDATS system. The software modules of LIDATS are configurable; they can be adjusted to harmonize data collection as well as transmission with the requirements of SUMO. Hence, our system can integrate with a SUMO simulation tool by using the TraCI protocol to make the exchange of data happen flawlessly. In addition, SUMO uses a unique XML schema for network data representation. So, the data would also have to be exported in the XML format from JSON with the help of a programming language, such as Python 3. Python 3 offers libraries that can parse JSON and create XML, allowing for a flexible and customized export process.

### 5.2. Commercial Traffic Simulations: Aimsun and VISSIM

Commercial simulations such as Aimsun and VISSIM have their own proprietary APIs and SDKs. Aimsun has the Aimsun Next API, which is intended for programmatic simulation data access. On the other hand, VISSIM offers a COM-based API, making it easier to control as well as exchanging data with the simulation environment. Our LIDATS system is engineered to interface with these APIs, allowing for the integration of real-time data into Aimsun and VISSIM. This interoperability plays a vital role in enhancing the simulation accuracy and the ability to reflect the current traffic conditions in a more reliable way in simulation scenarios.

### 5.3. Three-Dimensional Microscopic Traffic Simulations: PARAMICS

Three-dimensional traffic simulators like PARAMICS are very good at showing how each vehicle moves on the road in great detail. PARAMICS has special APIs that allow it to obtain live data from other systems. These APIs enable PARAMICS to work with real-time data systems such as our proposed model, LIDATS. This means that, as LIDATS collects data from traffic signal controller, it is capable of transmitting those data straight to the PARAMICS simulator using their APIs, and that makes the traffic simulations more realistic and up-to-date.

### 5.4. Advanced Tools: TransModeler

TransModeler is one of the advanced simulation tools. This tool raises the bar with specific interfaces, notably the Data Collector API, created to receive traffic data input. Specialized APIs like this one will smoothen the overall integration of real-time data streaming.

## 6. Digital Twin Benefits for Traffic Management

Transportation could become adaptive, responsive, and more efficient by using digital twin technology. This technology enables the real-time monitoring and simulation of the current traffic situation. It also provides a proactive approach to infrastructure maintenance as well as enhances the traffic optimization. This promises to be the next level of transportation planning and execution. As detailed, traffic management is much better when digital twins are involved. The benefits include the following:

1. Enhanced Decisionmaking: The digital twin technology will provide a detailed replica of the traffic ecosystem. It enables decisionmakers to visualize the impact of changes in near real time, predict the future conditions, as well as make informed choices. The precision in decisionmaking helps in reducing the approach of trial and error as well as ensuring that more effective traffic flow strategies are implemented.
2. Operational Efficiency: Digital twins assist in locating bottlenecks and streamlining the traffic patterns prior to making tangible alterations by simulating various traffic conditions. This ability to test solutions and refine them in a virtual environment paves the way for significant improvements in traffic flow and reduces road congestion.
3. Predictive Maintenance: The predictive capabilities of digital twins enable anticipating major infrastructure breakdowns, as well as scheduling maintenance activities before a critical failure occurs.
4. Emergency Response and Planning: Digital twins can simulate the impact on the traffic network in the event of accidents or a natural disaster. They play a vital role in developing an effective response strategy. This readiness helps in reducing traffic disruptions as well as enhancing public safety.
5. Sustainability and Environmental Impact: The role of digital twin technology is quite important to measure the environmental effects of traffic control systems, which may subsequently lead to the implementation of traffic control policies. It can play a vital role in helping cities to reduce their carbon emissions and improve the air quality significantly. Digital twin technology could simulate results by using models that take into account various programs such as congestion pricing or promotions to use alternative transportation modes and hence possibly contribute to imagining a more sustainable future.

## 7. Future Work

In the future, we are planning to test, refine, and improve our LIDATS system further in several key areas:

1. Ongoing Extensive Testing, Validation, and Data Handling: We recognize the importance of the continued rigorous testing of our LIDATS in real-world scenarios with a focus on edge cases and considering various environmental factors as well as traffic conditions. The ongoing testing will play a vital role in helping us in identifying potential challenges and refining the system’s hardware and software aspects. Furthermore, we will make sure that our model can efficiently handle the massive amounts of data the system generates. In order to accomplish this, we will work on refining the data compression and the transmission methods, and make sure that the data are delivered promptly without sacrificing their quality or reliability.

2. Enhanced Security Measures: We will evaluate and update the security measures of our LIDATS system consistently to protect it against emerging threats and vulnerabilities. The enhanced security measures will include using advanced encryption methods as well as intrusion detection systems (IDS).

3. Integration with Traffic Signal Controllers: As previously discussed, one of our main goals is to incorporate LIDATS into the traffic signal controller infrastructure, enabling seamless integration with the existing traffic infrastructure. It is important to note that each traffic signal controller manufacturer has its own proprietary Object Identifier (OID). The OID is essential for retrieving accurate information from the traffic signal controllers. In order to ensure that we achieve successful integration, we need to work closely with the manufacturers as well as suppliers to have access to the necessary technical documentation and obtain the essential OIDs from them. Having the OIDs from the manufacturers will enable us to collect and interpret the data from the traffic signal controller accurately. In addition, it will enhance the adaptability and versatility of our LIDATS system.

4. In-depth Data Analysis: Since massive data will be retrieved from various intersections, our plan is to thoroughly study and analyze the aggregated data from traffic signal controllers, and this can be achieved by assessing the traffic patterns and detecting anomalies or irregularities in the data. It may be possible to improve traffic management systems by gaining insights into future areas through the analysis of traffic data. Furthermore, we will discover more about the factors that influence traffic flow, congestion, and safety by utilizing sophisticated data analytics techniques like time series analysis as well as clustering.

5. Digital Twin Approach—Integration with the SUMO Traffic Simulator: One of our main future goals is to integrate our design with the open-source SUMO simulator. In order to properly provide the SUMO with real-time traffic feeds, we need to identify the necessary changes to the data formats, communication protocols, and transmission rates. This can be achieved by using the digital twin concept as it will highlight our approach and provide real-time data to different simulation platforms. In addition, the digital twin model with the SUMO simulation enables us to create a synchronized virtual representation for the traffic network so the simulation can respond to changes in real time and forecast the traffic patterns with greater precision.

## 8. Conclusions

In this paper, we introduced an adaptive system for aggregating traffic signal data and providing real-time feeds to traffic simulation environments. The conducted tests using the Econolite traffic signal controller simulator demonstrated the effectiveness and reliability of our LIDATS method. LIDATS provides the groundwork necessary to bridge the gap between simulation and actual on-site traffic control, introducing real-time data into the simulation. Our method has successfully captured, processed, and transmitted real-time traffic data, enabling their storage in a database that can be used later for further analysis and in training machine learning models for traffic control. Our tests’ promising results show that our LIDATS system has the potential to enhance traffic management systems by providing real-time traffic signal data. As we continue to refine and improve the system in the areas outlined in the Future Work section, we believe that our approach will play a significant role in advancing the traffic control strategies in modern cities. 

## Figures and Tables

**Table 1:** Integration capabilities of LIDATS with traffic simulation platforms for digital twins.

Platform	API/SDK Available	Integration Approach
SUMO [32]	TraCI Protocol	XML Generation and TraCI Components
Aimsun [33]	Aimsun Next API	Acquisition Modules and API Access
VISSIM [34]	COM-based API	COM API Connection
PARAMICS [35]	API Hooks	Dynamic Data Input Hooks
TransModeler [36]	Data Collector API	Real-time Data Streaming

## Data Availability

The raw data supporting the conclusions of this article will be made available by the authors on request.
